# Pan‐cancer RNA‐seq data stratifies tumours by some hallmarks of cancer

**DOI:** 10.1111/jcmm.14746

**Published:** 2019-11-15

**Authors:** F. Graeme Frost, Praveen F. Cherukuri, Samuel Milanovich, Cornelius F. Boerkoel

**Affiliations:** ^1^ Sanford Imagenetics Sioux Falls SD USA; ^2^ Sanford School of Medicine University of South Dakota Sioux Falls SD USA; ^3^ Sanford Research Center Sioux Falls SD USA; ^4^ Pediatric Hematology and Oncology Sanford Children's Hospital Sioux Falls SD USA

**Keywords:** cancer, hallmarks of cancer, pan‐cancer, precision medicine, precision oncology, RNA‐seq, transcriptome, wgcna

## Abstract

Numerous genetic and epigenetic alterations cause functional changes in cell biology underlying cancer. These hallmark functional changes constitute potentially tissue‐independent anticancer therapeutic targets. We hypothesized that RNA‐Seq identifies gene expression changes that underly those hallmarks, and thereby defines relevant therapeutic targets. To test this hypothesis, we analysed the publicly available TCGA‐TARGET‐GTEx gene expression data set from the University of California Santa CruzToil recompute project using WGCNA to delineate co‐correlated ‘modules’ from tumour gene expression profiles and functional enrichment of these modules to hierarchically cluster tumours. This stratified tumours according to T cell activation, NK‐cell activation, complement cascade, ATM, Rb, angiogenic, MAPK, ECM receptor and histone modification signalling. These correspond to the cancer hallmarks of avoiding immune destruction, tumour‐promoting inflammation, evading growth suppressors, inducing angiogenesis, sustained proliferative signalling, activating invasion and metastasis, and genome instability and mutation. This approach did not detect pathways corresponding to the cancer enabling replicative immortality, resisting cell death or deregulating cellular energetics hallmarks. We conclude that RNA‐Seq stratifies tumours along some, but not all, hallmarks of cancer and, therefore, could be used in conjunction with other analyses collectively to inform precision therapy.

## 1 INTRODUCTION

Human cancers are classified based on anatomical, histopathological and molecular features. Hanahan and Weinberg posited cancer unifying changes in cell biology (hallmarks): resisting cell death, sustaining proliferative signalling, evading growth suppressors, activating invasion and metastasis, enabling replicative immortality and inducing angiogenesis.^1^ Now included are two ‘enabling’ hallmarks (genome instability and mutation, and tumour‐promoting inflammation) and two ‘emerging’ hallmarks (deregulating cellular energetics and avoiding immune detection).^1^, ^2^, ^3^ Aberrant signalling axes identify specific hallmarks in a given cancer.^1^ Treatments targeting each hallmark promise individualized therapies.^1^, ^2^, ^4^

Individualized cancer therapy requires identifying contributors to these hallmarks, that is targetable biochemical pathways and genetic interactions. Presently, this includes histopathological, DNA, cytogenic and proteomic analyses.^5^, ^6^, ^7^Gene expression analysis is currently used case‐by‐case to discover targets 8; there is no consensus framework for using such analyses across all cancer types in clinical care. Although previous studies of specific primary site cancers such as pancreatic 9 and breast cancers 10 identified transcriptomic subgroups, investigation of transcriptomic subgroups across all cancers is not well studied. Using The Cancer Genome Atlas (TGCA) 11 and FANTOM5 12 transcriptomic data sets, Kaczowski et al 13looked for primary site‐independent cancer subgroups by grouping cancers according to differential expression of individual transcripts initially in cultured cells and secondarily in tumours. This analysis found that cancers could be classified into molecular subtypes defined by expression of transcripts involved in DNA and biopolymer metabolism, tumour suppression, oxidoreductase activity and developmental or cell cycle signalling.

The work of Kaczowski et al led us to hypothesize that cancer‐associated mutations and epimutations alter expression of co‐correlated groups of genes independent of cancer type and are detectable primarily in cancer tissue by RNA‐Seq. We reasoned that assessing for co‐correlated groups of genes is arguably more sensitive to changes in expression of gene networks underlying biological processes than is identifying common processes among individual transcripts. To test this, we analysed gene expression profiles from the University of California, Santa Cruz (UCSC) Toil recompute of the TCGA, Therapeutically Applicable Research to Generate Effective Treatments (TARGET)^14^ and Genotype‐Tissue Expression (GTEx)^15^ data sets available on the Xena Platform.^16^ We found consistent stratification of cancers by signatures of T cell activation, NK‐cell activation, complement cascade, ATM, Rb, angiogenic, MAPK, ECM receptor and histone modification signalling.

## 2 MATERIALS AND METHODS

### 2.1 Data sources and processing

**Figure 1:**
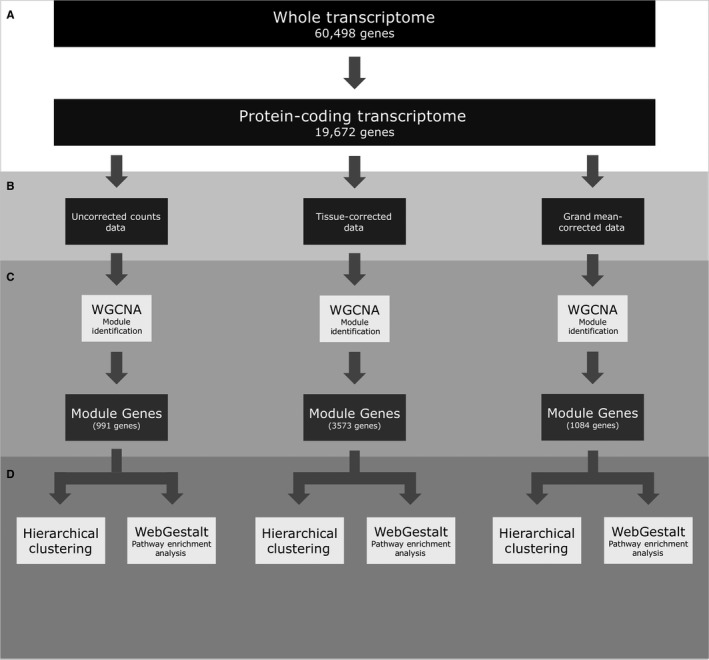
A flowchart depicting the analyses used in this study. Transcriptome profiles were first restricted to protein‐coding genes (A), then two different primary site‐correction approaches were taken to analyse the three data sets in parallel (B). Each data set was analysed using WGCNA to identify groups of genes (modules) that were co‐correlated, and variable across cancers (C). Genes found in modules were put through pathway enrichment analysis (WebGestalt) and used for hierarchical clustering (D)

The RNA‐Seq data and associated metadata files were downloaded from the UCSC Xena Data Browser^16^(2016‐09‐03 version, *TcgaTargetGtex_rsem_gene_tpm* (TTG) data set) (Table 1).These contained transcript‐non‐specific expression data for all coding genes as well as for long non‐coding RNA (lncRNA), pseudogenes and other non‐coding transcripts with unique Ensembl ENSG identifiers.^17^ The TTG data set quantifies gene expression as $\log_{2}\left(TPM+1\right)$ and were converted to TPM+1 for this analysis. The BioMart 18 database was used to extract genes having ENSG identifiers annotated with the *protein_coding* biotype. This eliminated 40,826 (67.5%) non‐coding entries leaving 19,672 protein‐coding entries (TTG‐C data set, Figure 1A, Table 1). The TTG‐C data set was then reduced to cancers that had corresponding normal samples and vice versa to create the T‐C‐PS and N‐C‐PS data sets, respectively (Table 1). Primary sites of uncertain histological equivalence between tumour and normal samples (eg blood cancers) or with sample numbers below 20 in either cancer or normal data sets were excluded.

**Table 1:** Characteristics of the data sets used in this study

Data set	Abbreviated name	Sample types	Number of samples	Number of primary sites	Number of genes
TcgaTargetGtex_rsem_gene_tpm	TTG	Tumour and normal	19,109	47	60,498*
TCGA‐TARGET‐GTEx_coding	TTG‐C	Tumour and normal	19,109	47	19,672
TTG_coding_common_primary	TTG‐C‐PS	Tumour and normal	12,166	15	19,672
Tumour_coding_common_primary	T‐C‐PS	Tumour	7,272	15	19,672
Normal_coding_common_primary	N‐C‐PS	Normal	4,894	15	19,672

* The 60,498 ‘genes’ in the TcgaTargetGtex_gene_expected_count data set includes various species of non‐coding RNAs and pseudogenes which have unique Ensembl ENSG identifiers.

### 2.2 Data normalization

Because non‐cancerous primary site‐specific gene expression might obscure cancer signatures, we used two methods to subtract non‐cancer expression data. We analysed data before and after correction (Figure 1B).

We used two binary primary site classification matrices: $P^{c}$, a t×q matrix of cancer primary sites, and $P^{n}$, a t×r matrix of normal tissue primary sites.* q* and *r* are the number of cancer and normal samples, respectively, and *t* is the number of primary sites. We used two gene expression matrices:*C,* a s×q matrix of cancer gene expression from the T‐C‐PS data set (Table 1), and N, a s×r matrix of normal tissue gene expression from the N‐C‐PS data set (Table 1).q and r are the number of cancer and normal samples, respectively, and s is the number of genes.

For a given cancer expression vector of gene i in matrix C, and for a binary classification vector for primary site l in matrix $P^{c}$, we derived the vector of tissue‐specific cancer gene expression $X_{i}$ by multiplying these two vectors:

$$X_{i}=P_{l}^{c}\times C_{i}$$

For a normal tissue, given the expression vector for gene i in matrix N and the binary classification vector for primary site l in matrix $P^{n}$, we derived the vector of tissue‐specific normal tissue gene expression $Y_{i}$ by multiplying these two vectors:

$$Y_{i}=P_{l}^{n}\times N_{i}$$

By calculating $X_{i}$ and $Y_{i}$ or all primary sites and all genes, we created a series of vectors that form the two three‐dimensional matrices X and Y. $X_{i,j,l}$ is the TPM gene expression value for gene i in cancer j of primary site l. $Y_{i,k,l}$ is the TPM gene expression value for gene i in normal tissue k of primary site l.

The tissue normal‐corrected data set (subsequently called ‘tissue‐corrected’) was calculated by first defining the mean normal expression ${\hat{G}}^{\text{tissue}}$ for gene i at each primary site l as:

$${\hat{G}}_{i,l}^{\text{tissue}}=\frac{1}{m_{l}}\sum_{k=1}^{r}Y_{I,K,L}$$

Where r is the number of normal tissue samples in primary site l, $m_{l}$ is calculated as:

$$m_{l}=\sum_{k=1}^{r}P_{k,l}^{n}$$

The tissue‐corrected gene expression matrix $L^{\text{tissue}}$ was calculated as:

$$L_{i,j,l}^{\text{tissue}}=\ln\left(\frac{X_{i,j,l}}{{\hat{G}}_{i,l}^{\text{tissue}}}\right)$$

The grand normal‐corrected data set (subsequently called ‘grand mean‐corrected’) was calculated by partitioning matrix Y by both the total number of primary sites, t and the number of cancers within each primary site, *r*:

$${\hat{G}}_{i}^{\mathrm{grand}}=\frac{1}{t}\sum_{l=1}^{t}\left(\sum_{k=1}^{r}\left(\frac{Y_{i,k,l}}{m_{l}}\right)\right)$$

$m_{l}$ was calculated as before. Finally, the grand mean‐corrected gene expression matrix $L^{\text{grand}}$ as calculated as:

$$L_{i,j}^{\text{grand}}=\ln\left(\frac{C_{i,j}}{{\hat{G}}_{i}^{\text{grand}}}\right)$$

### 2.3 Gene selection for clustering analysis

For clustering analysis, the genes profiled were restricted firstly to protein‐coding genes because mechanisms of tumorigenesis are currently better understood for the protein‐coding transcriptome (Figure 1A). Secondly, protein‐coding genes were restricted to ‘modules’ with expression values co‐correlated and variable across cancers using weighted gene co‐expression network analysis (WGCNA, Figure 1C).^19^ WGCNA was carried out using the *WGCNA* package in R (version 1.68).^20^ The mean TPM values of all genes in a module were used to evaluate the expression of a module in a cancer.

### 2.4 Characterization of modules identified by WGCNA

Modules were characterized using the over representation analysis (ORA) in the *WebGestaltR* package (version 0.4.1, Figure 1C).^21^ ORA used all protein‐coding genes as a reference set, the WikiPathway 22 database for functional annotations and the Benjamini‐Hochberg method 23 for multiple testing correction. Modules were named using default WGCNA settings, which assign each module a colour. The module names were not changed after characterization due to the complexity of the functional enrichment.

### 2.5 Clustering by transcript profiling

Clusters of similar cancers were defined by hierarchical clustering 24 using the cosine distance 25 between the expression profiles of the genes included in the modules and Ward's method 26 for agglomeration (Figure 1C). The number of clusters was determined with the *find_k* function from the *dendextend* R package (version 1.12.0); this function estimates *k* using maximal average silhouette widths.^27^ Dendrograms were cut into *k* groups to assign cancers to a cluster.

## 3 RESULTS

To test the hypotheses that cancer‐inducing gene expression changes are detectable by RNA‐Seq and traverse cancer primary sites, we analysed the TTG data set (Table 1) and restricted it to tumour and corresponding normal data (Figure 1; Table 1). These data sets were normalized, analysed and stratified.

### 3.1 Hallmark cancer and tissue‐specific pathways distinguish cancer clusters in uncorrected data

Analysis of the uncorrected data set showed that subtle expression differences in both hallmark cancer and cancer‐unrelated, tissue‐specific pathways differentiated clusters. WGCNA categorized 991 genes into 17 modules. Eleven of those modules were enriched for functional pathway annotations: brown, cyan, green, grey60, light yellow, midnight blue, pink, purple, red, tan and turquoise. Eight modules were enriched for tissue‐specific processes: brown, cyan, green, light yellow, pink, red, tan and turquoise (Table S1, ORA, *P* ≤ .049). The remaining three modules were enriched for cancer‐relevant processes. The grey60 and purple modules were, respectively, enriched for natural killer (NK) cell signalling and T cell receptor (TCR) signalling (Table S1, ORA, *P* ≤ 2.9*10^‐5^), axes characteristic of the avoiding immune destruction hallmark. The midnight blue module was enriched for histone modification signalling (Table S1, ORA, *P* ≤ 10^‐12^), a component of the genome instability and mutation hallmark.^1^

**Figure 2:**
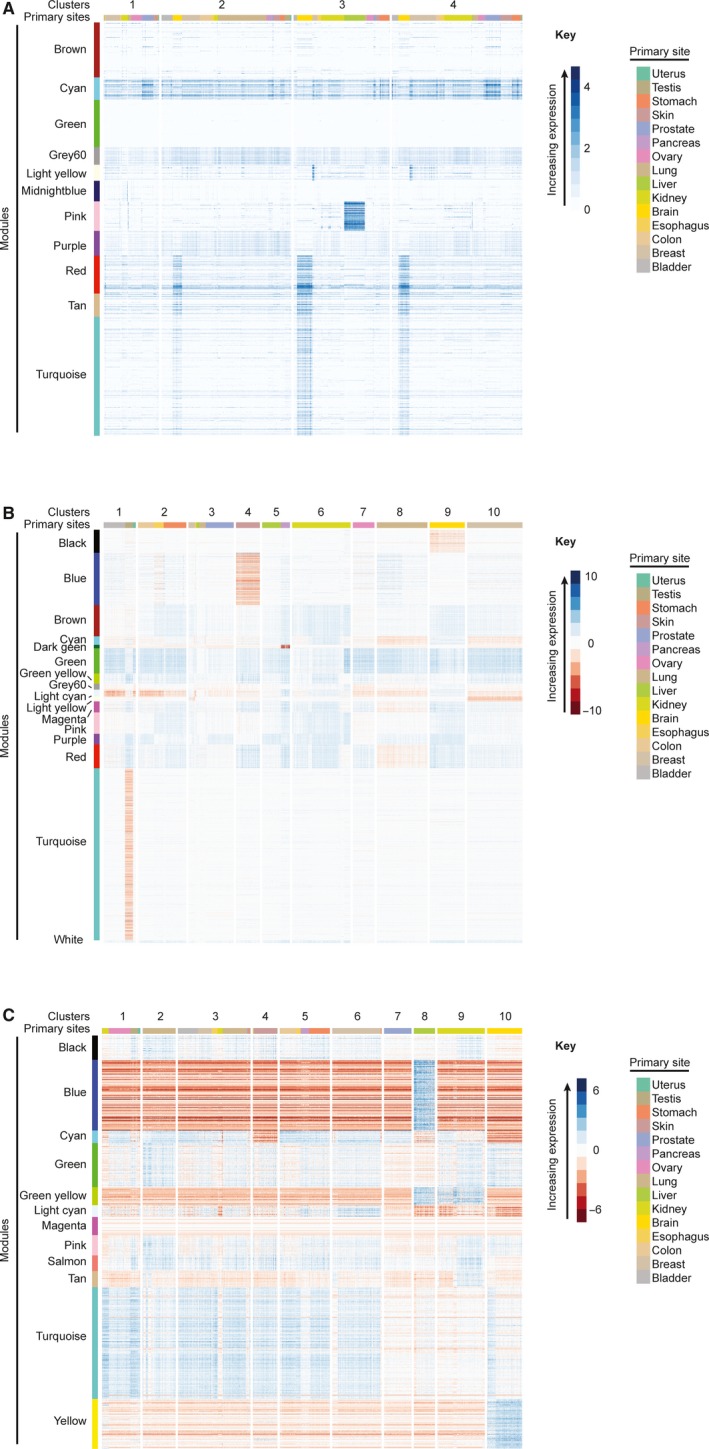
Heat maps of module expression within cancer clusters. Heat maps are shown for (A) uncorrected, (B) tissue‐corrected and (C) grand mean‐corrected RNA‐Seq data. WGCNA‐identified modules (left colour bar) are composed of protein‐coding genes with TPM values co‐correlated and variable across cancers. For panel A, module expression is the mean of TPM + 1 values for all genes within a module. For panels B and C, module expression is ln(Tumour/Normal) as defined in the Methods. Clusters of similar tumours (numbered divisions across the top) were defined by hierarchical clustering using the cosine distance between the genes included in the modules and Ward's method for agglomeration. The anatomical primary sites of tumours are graphically portrayed by the colour bar along the top

Hierarchical clustering of the 991 genes in WGCNA modules resulted in four cancer clusters. Each cluster was characterized by significantly different expression of the cyan, grey60, light yellow, midnight blue, pink, purple, red, tan and turquoise modules (Figure 2A, Kruskal‐Wallis Test, *P* ≤ 10^‐16^). The brown and green modules did not show differential expression (Figure 2A, Kruskal‐Wallis Test, *P* ≥ .35). Post hoc analysis by Dunn's Test for pairwise differences in module expression between clusters showed significantly different expression for four of six pairwise cluster comparisons for the turquoise module, five of six comparisons for the purple, red and tan modules, and six of six comparisons for the cyan, grey60, light yellow, midnight blue and pink modules (Table S2).

**Figure 3:**
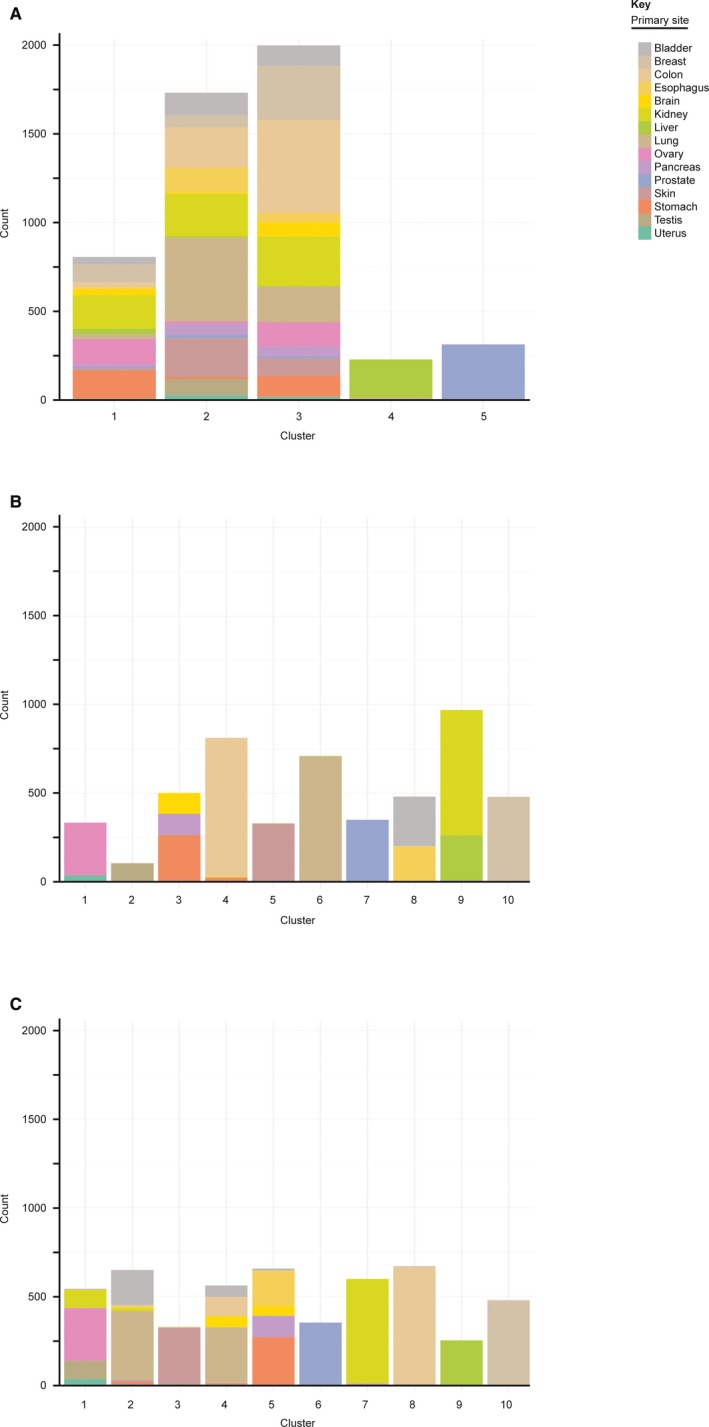
Graphs showing the distribution of each cancer type among clusters for (A) uncorrected, (B) tissue‐corrected and (C) grand mean‐corrected RNA‐Seq data. Count represents the number of tumours. Colours within the bars represent the cancer anatomical primary sites as described in the key

To investigate whether anatomical cancer primary site corresponded with cluster assignment, we evaluated the primary site composition of each cluster. All clusters were primary site heterogeneous. Cluster 1 was primarily composed of breast (26%), prostate (20%), ovary (18%) and kidney (12%) cancers (Figure 3A). Cluster 2 was predominantly composed of lung (37%) and breast (14%) cancers (Figure 3A). Cluster 3 was primarily composed of kidney (24%), liver (21%) and brain (16%) cancers (Figure 3A). Cluster 4 was primarily composed of kidney (21%), breast (20%) and prostate (11%) cancers (Figure 3A).

**Figure 4:**
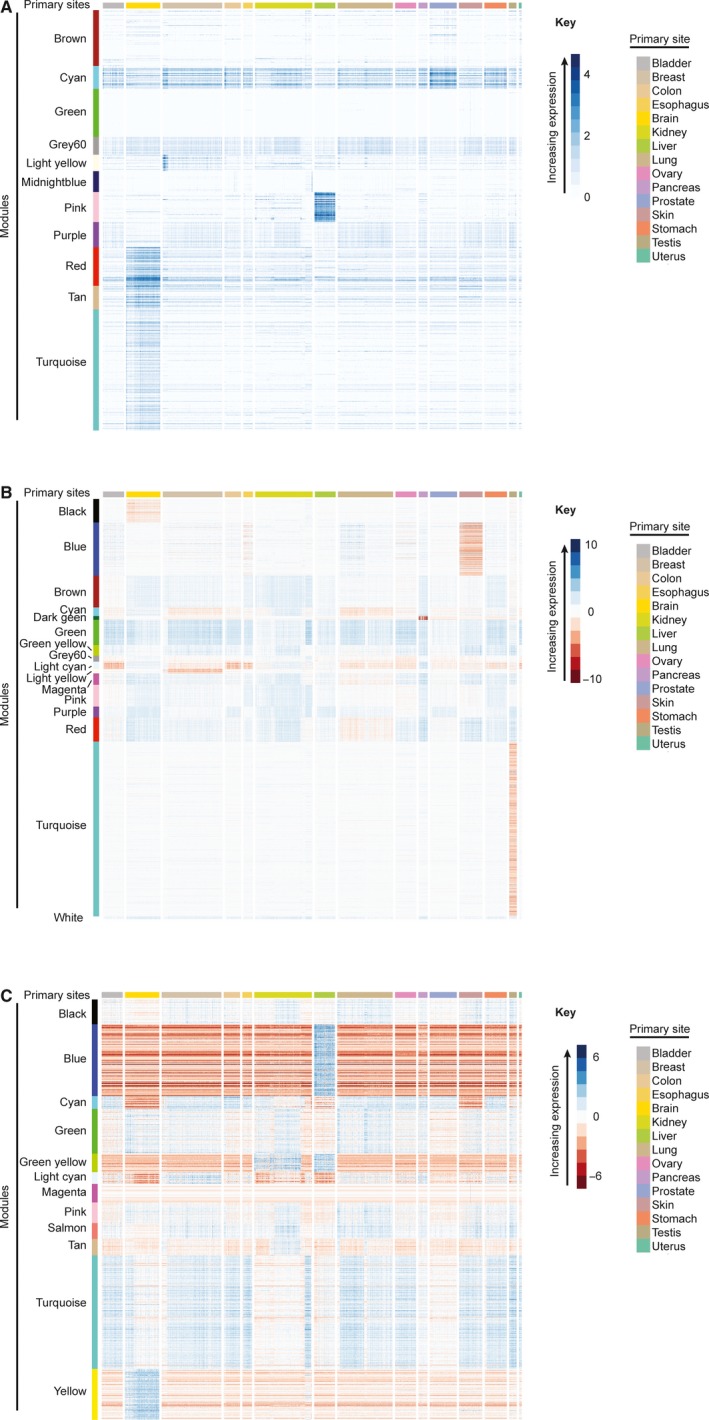
Heat maps of the module expression for each anatomical primary site (colour bar along the top).Heat maps are shown for (A) uncorrected, (B) tissue‐corrected and (C) grand mean‐corrected RNA‐Seq data. A heat map showing expression of modules (vertical colour bar) identified by WGCNA for each anatomical primary site (horizontal colour bar). WGCNA‐identified modules (left colour bar) are composed of protein‐coding genes with TPM values co‐correlated and variable across cancers. For panel A, module expression is the mean of TPM + 1 values for all genes within a module. For panels B and C, module expression is ln(Tumour/Normal) as defined in the Methods

Post hoc analysis by Tukey's Test determined pairwise differences in module expression between primary sites (Table S3). The brown module was expressed higher in uterine cancers than other sites (Figure 4A, Tukey HSD, *P* ≤ 3.2*10^‐9^). The light yellow module was expressed higher in breast cancers than other sites (Figure 4A, Tukey HSD, *P* ≤ 5.9*10^‐6^). The pink module was expressed higher in liver cancers than other sites (Figure 4A, Tukey HSD, *P* ≤ 2.0*10^‐11^). The red, tan and turquoise modules were expressed higher in brain cancers than other sites (Figure 4A, Tukey HSD, *P* ≤ 2.0*10^‐11^).The cyan module was expressed higher in stomach and prostate cancers than other sites (Figure 4A, Tukey HSD, *P* ≤ 7.6*10^‐6^). The green module was expressed higher in skin cancers than in prostate, liver, kidney, brain or breast cancers (Figure 4A, Tukey HSD, *P* ≤ .037).The expression of the grey60, midnight blue and purple modules differed between pairs of primary sites but without an appreciable pattern (Figure 4A, Tukey HSD, *P* ≤ .05).

### 3.2 Hallmark cancer and tissue‐specific pathways distinguish cancer clusters in tissue‐corrected data

Because of the potential for cancer‐unrelated primary site‐specific pathways to obfuscate tumorigenic signatures, we repeated the analyses (Figure 1D) after correcting for tissue‐specific gene expression. This removed some, but not all, of the primary site‐specific pathways seen in the uncorrected data and introduced new primary site‐specific pathways (Table S4 vs Table S1).

WGCNA identified 3573 genes distributed into 27 modules in the tissue‐corrected data. Of those modules, 16 were enriched for functional pathway annotations: black, blue, brown, cyan, dark green, green, green yellow, grey60, light cyan, light yellow, magenta, pink, purple, red, turquoise and white (Table S4, ORA, *P* ≤ .05). Of these 16 modules, 7 were enriched for tissue‐specific processes: black, blue, cyan, dark green, light cyan, light yellow and turquoise (Table S4). The remaining 9 modules were enriched for cancer‐relevant processes. The grey60, white and purple modules were enriched for mRNA splicing and translation (Table S4, ORA, *P* ≤ .004), processes globally dysregulated in cancer,^28^ although not a Hanahan and Weinberg described hallmark. The brown module was enriched for EGFR signalling (Table S4, ORA, *P* ≤ .05) and genes corresponding to mitogenic signalling axes (*BRAF*, *ERK*, *CREB1*, *JAK2* and *SOS2*(Table S4)); components of the sustained proliferative signalling hallmark. The pink module included genes involved in mitogenic signalling axes (*RICTOR*, *SOS1*, *MEKK2* and *REL*)(Table S4) and in histone modification (*ARID4B*, *KAT6A*, *KDM6A*, *TET2*, *KMT2C*, *ASH1L* and *KMT2E*)(Table S4); the former represents the sustained proliferative signalling hallmark and the latter the genome instability and mutation hallmark.^1^ The green module was enriched for processes related to cell cycle progression (Table S4), a characteristic of the evading growth suppressors hallmark. The green yellow, magenta and red modules were enriched for NK cell, T cell or inflammatory signalling (Table S4, ORA,*P* ≤ .05), markers of the tumour‐promoting inflammation and evading immune destruction hallmarks.

Hierarchical clustering of the 3573 genes in WGCNA modules detected 10 clusters characterized by distinct expression of 10 modules (Figure 2B, Kruskal‐Wallis Test, *P* ≤ 2.2*10^‐16^). Post hoc analysis by Dunn's Test to assess pairwise differences in module expression showed differential expression for 38 of 45 cluster comparisons for the blue and purple modules, 39 of 45 comparisons for the turquoise module, 40 of 45 comparisons for the black and dark green modules, 41 of 45 comparisons for the cyan, green yellow, grey60, light cyan and light yellow modules, 42 of 45 comparisons for the brown, green, magenta, red and white modules, and 44 of 45 comparisons for the pink module (Table S5). The high proportion of pairwise cluster comparisons with significant difference reinforces the distinctive expression patterns of each module across clusters.

To investigate whether anatomical cancer primary site corresponded with cluster assignment, we evaluated the primary site composition of each cluster. Clusters 1, 2, 3 and 5 were primary site heterogeneous. Cluster 1 was primarily composed of bladder (66%), testis (24%) and uterine (9%) cancers (Figure 3B). Cluster 2 was composed of stomach (47%), colon (33%) and oesophagus (20%) cancers (Figure 3B). Cluster 3 was predominantly composed of prostate (61%), lung (14%) and breast (13%) cancers (Figure 3B). Cluster 5 was composed of liver (67%) and pancreas (33%) cancers (Figure 3B). Clusters 4, 6, 7, 8, 9 and 10 were ≥ 99.9% a single primary site: skin, kidney, ovarian, lung, brain and breast cancers, respectively (Figure 3B). The primary site homogeneity of clusters suggests either that correction for primary site signatures in this data set was incomplete or that the detected cancer signatures are primary site‐dependent.

Post hoc analysis by Tukey's Test determined pairwise differences in module expression between primary sites (Table S6). The expression of the black, blue, brown, cyan, dark green, green, green yellow, grey60, light cyan, light yellow, magenta, pink, purple, red, turquoise and white modules differed between primary sites but without an appreciable pattern (Figure 4B, Tukey HSD, *P* ≤ .05).

### 3.3 Hallmark cancer pathways primarily distinguish cancer clusters in grand mean‐corrected data

Given modules enriched for normal tissue processes in the tissue‐corrected data, we corrected for non‐cancer gene expression using the grand mean expression of each gene in all non‐cancer primary sites. This stratified tumours according to some hallmarks.

WGCNA identified 1084 genes in 17 modules, of which 12 were enriched for functional pathway annotations: black, blue, cyan, green, green yellow, light cyan, magenta, pink, salmon, tan, turquoise and yellow (Table S7, ORA, *P* ≤ .05). Of those 12 modules, 4 were enriched for tissue‐specific processes: blue, green yellow, magenta and yellow (Table S7, ORA, *P* ≤ .05). The remaining 8 modules were enriched for cancer‐relevant processes. The tan module was enriched for markers of angiogenesis (Table S7, ORA, *P* ≤ .01), that is the inducing angiogenesis hallmark. The turquoise module was enriched for cell cycle progression pathways (Table S7, ORA, *P* ≤ .03), a component of the evading growth suppressors hallmark. The cyan and light cyan modules were, respectively, enriched for markers of the epithelial to mesenchymal transition (EMT) and extracellular matrix (ECM) receptor and adhesion signalling (Table S7, ORA,*P* ≤ .02), components of the activating invasion and metastasis hallmark. The black, green, pink and salmon modules were enriched for NK cell, T cell or inflammatory signalling (Table S7, ORA,*P* ≤ .05), components of the tumour‐promoting inflammation and evading immune destruction hallmarks.

Hierarchical clustering of the 1,084 genes in grand mean‐corrected WGCNA modules defined 10 clusters. These clusters were characterized by distinct expression of modules (Figure 2C). All modules showed differential expression across clusters (Figure 2C, Kruskal‐Wallis Test, *P* ≤ 2.2*10^‐16^).

Post hoc analysis by Dunn's Test for pairwise differences in module expression showed differential expression for 31 of 45 cluster comparisons for the magenta module, 37 of 45 for the black module, 38 of 45 for the salmon module, 40 of 45 for the green yellow, pink and tan modules, 41 of 45 for the turquoise and yellow modules, 42 of 45 for the light cyan module, 43 of 45 for the blue and green modules, and 44 of 45 for the cyan module (Table S8).

To investigate whether anatomical cancer primary site corresponded with cluster assignment, we evaluated the primary site composition of each cluster. Clusters 1, 3 and 5 were primary site heterogeneous. Cluster 1 was primarily composed of ovarian, (56%), testis (19%) and kidney (16.8%) cancers (Figure 3C). Cluster 3 was predominantly composed of lung (33%), bladder (28%) and breast (18%) cancers (Figure 3C). Cluster 5 was primarily composed of stomach (42%), colon (33%) and pancreas (17%) cancers (Figure 3C).

Post hoc analysis by Tukey's Test determined pairwise differences in module expression between primary sites (Table S9). The green yellow module was expressed higher in kidney and liver cancers than other primary sites (Figure 4C, Tukey HSD,*P* ≤ 2.0*10^‐11^). The magenta module was expressed higher in skin cancers than in prostate, pancreas, ovary, lung, liver, kidney, brain, colon and breast cancers (Figure 4C, Tukey HSD,*P* ≤ .02). The expression of black, blue, cyan, green, light cyan, pink, salmon, tan, turquoise and yellow modules differed for many pairwise comparisons of primary sites but did not have an appreciable pattern (Figure 4C, Tukey HSD, *P* ≤ .05).

### 3.4 Clusters are incompletely primary site‐independent

To evaluate whether cancer clusters express hallmarks independently of primary site, we assessed the stratification of a primary site across clusters and the primary site diversity within clusters. For the former, we counted the number of clusters in which the null hypothesis of the hypergeometric test was rejected (*P* ≤ .05, Table S10). Primary sites with 2 or more clusters that rejected that null hypothesis were considered stratified across clusters. We observed that in the uncorrected data breast, oesophagus, kidney, ovary, prostate, stomach and uterine cancers were stratified by their gene expression profiles (Table 2), that in the tissue‐corrected data no cancer types were stratified by their gene expression profiles (Table 2), and that in the grand mean‐corrected data breast, oesophagus and lung cancers were stratified by their gene expression profiles (Table 2).

**Table 2:** The number of clusters with more tumours from a primary site than expected by chance as determined by the hypergeometric test

Data set	Bladder	Brain	Breast	Colon	Oesophagus	Kidney	Liver	Lung	Ovary	Pancreas	Prostate	Skin	Stomach	Testis	Uterus
UC	1	1	2	1	2	2	1	1	2	1	2	1	2	1	2
TC	1	1	1	1	1	1	1	1	1	1	1	1	1	1	1
GC	1	1	2	1	2	1	1	2	1	1	1	1	1	1	1

Abbreviations: UC, uncorrected data; TC, tissue‐corrected data; GMC, grand mean‐corrected data

## 4 DISCUSSION

By WGCNA, hierarchical clustering and ORA analyses, RNA‐Seq detects gene expression changes contributing to some cancer hallmarks. Of the ten hallmarks identified by Hanahan and Weinberg, our analyses detected modules enriched for seven: evading growth suppressors, tumour‐promoting inflammation, avoiding immune destruction, inducing angiogenesis, sustained proliferative signalling, activating invasion and metastasis, and genome instability and mutation. The gene expression changes corresponding to these hallmarks stratify a subset of cancers across clusters, although not fully independent of tumour primary site.

### 4.1 RNA‐Seq data consistently stratifies cancers by seven therapeutically targetable hallmarks of cancer

Consistent with prior studies,^29^ our analyses showed enrichment for pathways representative of the sustained proliferative signalling hallmark. The common perturbation of mitogenic signalling axes such as MAPK or PI3K‐Akt‐mTOR has led to development of inhibitors of those axes, although those inhibitors have not been potent single‐agents,^30^, ^31^ therapies targeting cell cycle progression, the end‐point of proliferation cascades commonly activated in cancer,^29^, ^32^ are being actively investigated. CDK4/6 inhibitors have shown efficacy in trials for late stage breast and lung cancers.^33^, ^34^

The detection of modules enriched for cell cycle pathways represent the evading growth suppressors hallmark. Expression of genes such as *CDKN2A*, *CCNE1* and *RB1*, which are all components of cell cycle signalling, has been implicated in resistance to CDK4/6 inhibitors.^33^This illustrates that expression assays might have utility detecting biomarkers for resistance to therapies targeting the sustained proliferative signalling hallmark.

Components of the tumour‐promoting inflammation and the avoiding immune destruction hallmarks were enriched in several modules and were detected in all data sets (Tables S1, S4, S7).Because only a minority of patients within a given cancer type respond to CD8^+^T cell dependent cancer immunotherapy,^35^induction of CD8^+^T cell recruitment and activation 36or inclusion of innate immune processes such as NK‐cell activation are being developed.^37^ This expansion requires characterization of biomarkers defining an antitumoral immune response. The presence of T cell activation, NK‐cell activation and inflammatory signalling axes in our analysis suggests that gene expression assays might contribute such biomarkers.

Our analyses detected modules enriched for angiogenesis hallmark related processes. Although clinical targeting of the VEGF signalling axis frequently induces resistance^38^ that limits it as a monotherapy, expression of VEGF and its receptors correlates with cancer stage and metastasis and might be a useful prognostic indicator.^39^ The variation in the angiogenic signalling detected in our study divides breast, kidney and colon cancers into high and low expression groups (Figure 4C), a division that might not only be useful as a staging marker but also for identifying tumours likely to respond to antiangiogenic therapy.

Detection of markers of EMT and ECM signalling axes processes of the activating invasion and metastasis hallmark suggests that gene expression assays might contribute to the individualization of future antimetastatic drug cocktails. Despite limitations, there are current therapeutic strategies to inhibit the metastatic potential including targeting VEGF, the NF‐κB pathway and integrin signalling.^40^

Chromatin remodelling pathways, which we detected, are both components of the genome instability and mutation hallmark^1^ and therapeutic targets. Inhibitors of chromatin remodelling have been in use for over a decade, although primarily for leukemias and lymphomas.^41^ Because nearly half of cancers have alterations of chromatin remodelling, several current trials target aspects of chromatin remodelling in solid cancers.^41^ Grouping cancers by their precise mechanisms of dysregulated chromatin remodelling assists selecting appropriate therapies, and our analyses suggest gene expression assays might assist with this.

The detection of multiple hallmarks by gene expression analyses highlights a potential for RNA‐Seq to identify therapeutic combinations as our analyses subgrouped some cancers according to expression of multiple modules. For example, kidney cancers subdivided into three subgroups: (a) high expression of genes enriched for TCR signalling and for angiogenic signalling, (b) low expression of both TCR signalling and angiogenic signalling genes, and (c) high expression of TCR signalling and low expression of angiogenic signalling genes (Figure 4C). Although not yet tested clinically, such subgroups could provide useful as biomarkers for multimodal treatment of kidney cancer given that immunotherapy and antimetastatic drugs would theoretically target subgroup 1, standard chemotherapies would target subgroup 2 and immunotherapies would target subgroup 3.

Multimodal treatment targets multiple hallmarks concurrently because the strong selective pressure on cancer cell populations 42leads to resistance to monotherapies.^43^Additional multimodal therapies include dual inhibition of the mitogenic and cell cycle signalling pathways,^44^CDK4/6 inhibitors plus immunotherapy 45 and VEGF inhibition plus multiple classes of antimetastatic therapies,^40^ which, respectively, correspond to the sustained proliferative signalling, evading growth suppressors, inducing angiogenesis, and activating invasion and metastasis hallmarks of cancer. The analyses herein subdivided some tumours according to combination of these hallmarks.

### 4.2 RNA‐Seq data does not stratify cancers by three hallmarks of cancer

Our analyses did not detect three of Hanahan and Weinberg's hallmarks as gene expression modules stratifying tumours. These hallmarks were resisting cell death, deregulating cellular energetics and enabling replicative immortality.

Strong transcriptomic signatures are not expected for the sustained enabling replicative immortality hallmark. This hallmark is predominantly characterized by the expression of *TERT*.^1^, ^46^ *TERT* alone is insufficient as a transcriptomic network to be detected by our analyses.^20^

The deregulating cellular energetics hallmarks have a strong transcriptomic footprint.^1^, ^47^ The processes underlying this hallmark originate from changes in gene expression, namely, glucose transport,^48^ glutamine transport^49^ and the pentose phosphate pathway,^50^ as well as the biosynthesis of nucleotides,^51^ serine,^52^ arginine 53 and proline.^54^ Studies detecting these changes in gene expression, however, either did not use tumour biopsies as the source tissue or use more targeted methods than RNA‐Seq. In contrast to cultured tumour cells or xenografts of cultured tumour cells, our analyses agnostically probed tumour biopsies, a highly complex cell population,^55^ for gene expression signatures varying across samples. Consequently, we postulate that tissue heterogeneity introduces too much biological variability or that our assumption of variability across cancers is invalid. Supporting the latter hypothesis, prior studies show consistent expression of metabolic genes across cancer types,^56^ and we find that genes with the least variable expression are enriched for metabolic pathway annotations (Table S11).

Like the cellular energetics hallmark, the resisting cell death hallmark has a strong transcriptomic footprint. It is characterized by the subversion of the regulatory and functional elements of the cellular apoptosis machinery.^1^ Cancer cells impair apoptosis by decreasing expression of proapoptotic proteins or by increasing expression of antiapoptotic proteins.^57^ The specific family members up or down‐regulated are, however, relatively cancer type‐specific.^57^ Consequently, although gene expression networks involved with apoptosis are altered, specific cancers usually have changes in only a few genes in that network,^57^ and our methodology is insensitive to such limited changes.

### 4.3 Clusters defined by hallmark gene expression are incompletely independent of cancer primary sites

Analyses of the uncorrected data set showed that brain, oesophageal, ovarian, prostate, stomach and uterine cancers were stratified across clusters (Table 2). Due to the presence of modules enriched for non‐cancer processes (Table S1) and the lack of distinct expression of modules enriched for hallmarks across clusters (Figure 2), we are not certain that hallmark cancer signatures solely underlie that stratification.

Suggesting the dependence of clustering on the anatomical primary site, analyses of the tissue‐corrected data set found that no cancer types were stratified across clusters (Table 2). The normalization for the tissue‐corrected data set used separate ‘normal’ gene expression vectors for each primary site and that process could introduce signatures by over‐correction. Supporting this, comparison to the uncorrected data shows the concurrent elimination of modules enriched for non‐cancer processes in the uncorrected data and the detection of other modules enriched for non‐cancer processes (Tables S1, S4).

Analyses of the grand mean‐corrected data set showed that breast, oesophageal and lung cancers were stratified across clusters (Table 2), suggesting that clusters are incompletely independent of primary site. Although there are four modules enriched for non‐cancer processes, the expression of those modules only distinguishes three of ten clusters (Figure 2C). The modules enriched for hallmarks are the primary differentiators of clusters responsible for stratifying cancers. This stratification is unlikely to be an artefact of the normalization process since the use of a uniform ‘normal’ gene expression vector for all cancers could introduce consistent signatures that would not be considered in module detection by WGCNA. This raises the promise that clustering by expression of genes relevant to cancer hallmarks stratifies cancers to provide prognostically relevant information or therapeutically relevant information, particularly in conjunction with histopathologic, DNA or proteomic data.

### 4.4 Biological processes identified in this study align with previous literature

Previous investigations into transcriptomic subdivisions of cancer observed several of the pathways that we identified. Specifically, pancreatic,^9^ breast 10 and pan‐cancer 13 studies found that cell cycle pathways define transcriptomic subgroups and that immune signalling defines subgroups of pancreatic 9 and breast 10 cancers. In contrast to the pan‐cancer study of Kaczowski et al did,^13^ our analysis did not detect differential expression of genes relevant to the cellular energetics hallmark; we hypothesize, as discussed above, that this arose because our analysis of co‐correlated groups of transcripts is insensitive to small numbers of transcripts with altered expression, whereas the approach of Kaczowski et al is not so limited because it focuses on differential expression of individual transcripts.^13^ On the other hand, detecting expression changes in the chromatin remodelling, angiogenesis and ECM signalling axes, our analysis distinguished molecular subtypes that were not noted in studies of the pancreatic,^9^ breast 10 or pan‐cancer 13 data sets.

### 4.5 Limitations

The detection of some cancer hallmark signatures is encouraging, given the conservative approach (requiring an expression signature across all cancers) and the following limitations of our study. First, the data set did not contain isoform‐specific expression, and this prevented the incorporation of alternatively spliced transcripts into our analysis. Alternatively, spliced transcripts play important roles in cancer cell biology 58 and are relevant to all hallmarks of cancer.^28^ Second, although dysregulation of non‐coding RNAs is integral to cancer biology 59 and play a therapeutically relevant role,^60^ we restricted our analysis to the protein‐coding transcriptome to facilitate pathway enrichment analysis. Third, our analysis did not account for therapeutically relevant gene fusions detectable by RNA‐Seq.^61^ Fourth, we did not assess allele‐specific expression, which is relevant to cancer biology and progression.^62^ Fifth, to mitigate the effects of spurious expression differences, we did not consider modules of single or small numbers of genes^20^; this might be addressed in the future by the inclusion of spike‐in reference RNAs.^63^ Sixth, for several technical reasons, our analysis was indifferent to tumour microenvironments (TMEs) which are known to modify processes (proliferation, metastasis and interaction with immune cells 64) underlying cancer hallmarks. Although these limitations decreased the sensitivity of our analysis to hallmark changes, they do not alter the specificity of our analysis.

## 5 CONCLUSIONS

RNA‐Seq detects some hallmarks of cancer and those hallmarks stratified some, but not all, cancer types. We consistently identified signatures corresponding to the tumour‐promoting inflammation and avoiding immune destruction hallmarks (T cell activation, NK‐cell activation and complement cascade activation), the evading growth suppressors (ATM, Rb and G1 to S phase transition signalling), the inducing angiogenesis (angiogenesis signalling), the sustained proliferative signalling (*BRAF*‐*ERK*‐*CREB1*), the activating invasion and metastasis (ECM receptor signalling and EMT markers), and the genome instability and mutation (histone modification) hallmarks. Additionally, cancer clusters differentiated by the above hallmarks stratified breast, oesophageal and lung cancers, highlighting the possibility of targeting transcriptomic features independent of anatomical primary site. Future studies are required to determine the therapeutic and prognostic relevance of these findings and to assess the impact of including transcriptomic features that we did not analyse.

## CONFLICTS OF INTEREST

The authors declare that the research was conducted in the absence of any commercial or financial relationships that could be construed as a potential conflict of interest.

## AUTHOR CONTRIBUTIONS

CB conceived the hypothesis tested in this study. GF and PC developed the methodology to test that hypothesis. All code for data analysis and processing was developed by GF. GF wrote the manuscript with input and revisions provided by CB, PC and SM. SM and CB aided the clinical and biological relevance of conclusions drawn from data analysis.

## Data Availability

All data used in this study is freely available from the UCSC Xena Data Browser. Specifically, the TCGA‐TARGET‐GTEx gene expression data set, along with associated metadata files, is available at the following link: https://xenabrowser.net/datapages/?dataset=TcgaTargetGtex_rsem_gene_tpm&host=https%3A%2F%2Ftoil.xenahubs.net&removeHub=https%3A%2F%2Flocal.xena.ucsc.edu%3A7223
