# Supplementary material for: Pan‐cancer RNA‐seq data stratifies tumours by some hallmarks of cancer
Source: J Cell Mol Med. 2019 Nov 15;24(1):418–30. doi: 10.1111/jcmm.14746 (PMC6933344; doi:10.1111/jcmm.14746)
Supplement: Supplementary file 1 [file JCMM-24-418-s001.docx]

**Pan-Cancer RNA-Seq Data Stratifies Tumors by Some Hallmarks of Cancer**

**F. Graeme Frost^1^, Praveen F. Cherukuri^1,2,3^, Samuel Milanovich^2,3,4*^, Cornelius F. Boerkoel^1^**

^1^ Sanford Imagenetics, Sioux Falls, SD, USA

^2^ Sanford School of Medicine, University of South Dakota, Sioux Falls, SD, USA

^3^Sanford Research Center, Sioux Falls, SD, USA

^4^Division of Pediatric Hematology and Oncology, Sanford Children’s Hospital, Sioux Falls, SD, USA

**Supplemental Methods**

## *Statistical analyses*

To evaluate differences in mean expression of genes in each module across clusters and across primary sites, each comparison was evaluated for normality using the Shapiro-Wilk test of normality [1] and for equal variance using Levene’s test of homogeneity of variance across groups [2]. If Shapiro-Wilk test H_0_ was not rejected (*P*≥0.05; *H_0_*: normal distribution) and Levene’s test H_0_ was also not rejected (*P*≥0.05; H_0_: equal variance across groups), then Analysis of variance (ANOVA)[3] was used. If the ANOVA null hypothesis that variance in the data are due to chance was rejected (*P*≤0.05), Tukey’s range test [4] was used for *post-hoc* analysis. If either the Shapiro-Wilk test null hypothesis that data are normally distributed or Levene’s test null hypothesis that there is equal variance across groups was rejected (*P*≤0.05), then the Kruskal-Wallis test [5] was used to compare median module expression across clusters and across primary sites. If the Kruskal-Wallis null hypothesis that distributions across groups was the same was rejected (*P*≤0.05), then Dunn’s test [6] was used for *post-hoc* analysis. To test whether the number of cancers of a given primary site in each cluster was greater than expected by chance, a one-tailed hypergeometric test was used.

## *Computational resources*

All computational tasks in this analysis were implemented using a computer with an 8 core 2.9 GHz Intel Core i7 processor and 16GB RAM. Statistical and graphical analysis and plotting were done using the R language and software environment [7]. R packages were used for hierarchical clustering, plotting, statistical tests, reading and writing files, and data manipulation (Supplementary materials).

*R packages used for data analysis and processing*

The following R packages were used for hierarchical clustering: *wordspace* (Version 0.2-5) [8], *dendextend* (Version 1.12.0) [9], and *flashClust* (Version 1.01-2) [10]. For plotting, we used the *pheatmap* (Version 1.0.12) [11], *ggplot2* (Version 3.2.0) [12], *RColorBrewer* (Version 1.1-2) [13], and *gridExtra* (Version 2.3) [14] packages. For Shapiro-Wilk test, Levene’s test, ANOVA, Tukey’s range test, the Kruskal-Wallis test, and Dunn’s test we used the *car* (Version 3.0-3) [15] and *DescTools* (Version 0.99.28) [16] packages. For reading and writing files we used the *data.table* (Version 1.12.2) [17] package. For data manipulation we used the *dplyr* (Version 0.8.3) [18], *magrittr* (Version 1.5) [19], and *stringr* (Version 1.4.0) [20] packages. All code used for this project is available at <https://github.com/fgfrost/PanCan_RNA-Seq>

*Characterization of the most and least variable genes in this dataset*

Genes were characterized as either overdispersed (relatively variable) or underdispersed (relatively unvariable) by calculating the Fano Factor for each gene:

| $Fano Factor = \frac{\sigma_{gene}^{2}}{\mu_{gene}}$ | (1) |
| --- | --- |

Where $\sigma_{gene}$ is the standard deviation of the expression a given gene, and $\mu_{gene}$ is the mean expression of a given gene. Fano Factor > 1 was considered overdispersed, and Fano Factor < 1 was considered underdispersed. Over- and underdispersed genes were then split into quartiles, and each quartile was tested for enrichment of annotated biological process gene ontology terms using the *topGO* package (Version 3.9) in R

**Supplemental Results**

**Table S1.** Full output of ORA of modules detected by WGCNA in the Uncorrected data.

This table is attached in the supplementary files as “Table_S1.csv”

**Table S2.** Post-hoc analysis of cluster-wise module gene expression of the uncorrected dataset, using Dunn’s test

This table is attached in the supplementary files as “Table_S2.csv”

**Table S3.** Post-Hoc analysis of tissue-wise module expression of the uncorrected dataset.

This table is attached in the supplementary files as “Table_S3.csv”

**Table S4.** Full output of ORA of modules detected by WGCNA in the Tissue-corrected data.

This table is attached in the supplementary files as “Table_S4.csv”

**Table S5.** Post-hoc analysis of cluster-wise module gene expression of the tissue-corrected dataset, using Dunn’s test.

This table is attached in the supplementary files as “Table_S5.csv”

**Table S6.** Post-Hoc analysis of tissue-wise module expression of the tissue-corrected dataset.

This table is attached in the supplementary files as “Table_S6.csv”

**Table S7.** Full output of ORA of modules detected by WGCNA in the Grand mean-corrected data

This table is attached in the supplementary files as “Table_S7.csv”

**Table S8** Post-hoc analysis of cluster-wise module gene expression of the grand mean-corrected dataset, using Dunn’s test

This table is attached in the supplementary files as “Table_S8.csv”

**Table S9.** Post-Hoc analysis of tissue-wise module expression of the grand mean-corrected dataset.

This table is attached in the supplementary files as “Table_S9.csv”

**Table S10.** *P* values from the hypergeometric test for all primary sites in each cluster, from each dataset.

This table is attached in the supplementary files as “Table_S10.csv”

**Table S11.** Analysis of functional enrichment of the most and least variable protein-coding genes across all cancers in the TcgaTargetGtex_gene_tpm dataset.

This table is attached in the supplementary files as “Table_S11.csv”

**Supplementary References**

[1] **Shapiro SS, Wilk MB**. An Analysis of Variance Test for Normality (Complete Samples). *Biometrika* 1965; 52; 591–611.

[2] **Levene H, Olkin II, Hotelling H**. Robust Tests for Equality of Variances. *Contributions to Probability and Statistics; Essays in Honor of Harold Hotelling* 1960; 78–92.

[3] **Fisher RA**. On the probable error of a coefficient of correlation deduced from a small sample. *Metron* 1921; 1; 3–32.

[4] **Tukey JW**. Comparing Individual Means in the Analysis of Variance. *Biometrics* 1949; 5; 99–114.

[5] **Kruskal WH, Wallis WA**. Use of Ranks in One-Criterion Variance Analysis. *Journal of the American Statistical Association* 1952; 47; 583–621.

[6] **Dunn OJ**. Multiple Comparisons Using Rank Sums. *Technometrics* 1964; 6; 241–52.

[7] **Team RC**. R: A Language and Environment for Statistical Computing. Vienna, Austria: R Foundation for Statistical Computing; 2019.

[8] **Evert S**. Distributional Semantics in R with the wordspace Package. Proceedings of COLING 2014, the 25th International Conference on Computational Linguistics: System Demonstrations, Dublin, Ireland: 2014 p.110–114, p. 110–114.

[9] **Galili T**. dendextend: an R package for visualizing, adjusting and comparing trees of hierarchical clustering. *Bioinformatics* 2015; 31; 3718–20.

[10] **Langfelder P, Horvath S**. Fast R Functions for Robust Correlations and Hierarchical Clustering. *Journal of Statistical Software* 2012; 46; 1–17.

[11] **Kolde R, Kolde MR**. Package ‘pheatmap.’ *R Package* 2015; 1.

[12] **Wickham H**. ggplot2: Elegant Graphics for Data Analysis. Springer-Verlag New York; 2016.

[13] **Neuwirth E**. RColorBrewer: ColorBrewer Palettes. 2014.

[14] **Auguie B**. gridExtra: Miscellaneous Functions for “Grid” Graphics. 2017.

[15] **Fox J, Weisberg S**. An R Companion to Applied Regression. Second. Thousand Oaks CA: Sage; 2011.

[16] **al AS et mult**. DescTools: Tools for Descriptive Statistics. 2019.

[17] **Dowle M, Srinivasan A**. data.table: Extension of `data.frame`. 2019.

[18] **Wickham H, François R, Henry L, et al.** dplyr: A Grammar of Data Manipulation. 2019.

[19] **Bache SM, Wickham H**. magrittr: A Forward-Pipe Operator for R. 2014.

[20] **Wickham H**. stringr: Simple, Consistent Wrappers for Common String Operations. 2019.
